# Association between resolved hepatitis B virus infection and femoral and spinal bone mineral density in American adults: a cross-sectional study

**DOI:** 10.3389/fendo.2023.1237618

**Published:** 2023-09-27

**Authors:** Yan Yang, Jing Zeng, Tingting Zhang, Jinjing Wang, Xiaojing Fan, Qiaomin Wang, Xuan Wang, Zhengrong Qi, Yi Fang

**Affiliations:** ^1^ Department of Endocrinology, The Fifth Medical Center of PLA General Hospital, Beijing, China; ^2^ Department of Orthopedics, Beijing Friendship Hospital, Capital Medical University, Beijing, China

**Keywords:** resolved hepatitis B virus infection, femoral bone mineral density, spinal bone mineral density, adults, NHANES

## Abstract

**Background:**

Hepatitis B virus (HBV) infection is a global health concern that can potentially affect bone health. However, the specific association between resolved HBV infection and bone mineral density (BMD) remains unclear. This cross-sectional study aimed to investigate the potential association between resolved HBV infection and femoral and spinal BMD in adults in the United States.

**Methods:**

This cross-sectional study included participants aged 20-79 years with negative HBV surface antigen (HBsAg) from the 2005-2010, 2013-2014, and 2017-2018 cycles of the National Health and Nutrition Examination Survey. Resolved HBV infection was defined as negative HBsAg with positive HBV core antibody. BMD was measured using dual-energy X-ray absorptiometry. Propensity score matching (PSM) was performed to balance baseline characteristics.

**Results:**

A total of 10,333 eligible participants were identified and matched, of whom 737 (7.1%) had resolved HBV infection. Men with resolved HBV infection had significantly lower femoral and spinal BMD compared to those with no HBV infection, both before and after PSM. In the matched population, resolved HBV infection in men was negatively associated with femoral BMD (β= -0.024, 95% CI: -0.047 to -0.002, p = 0.0332) and spinal BMD (β= -0.025, 95% CI: -0.048 to -0.002, p = 0.0339). Postmenopausal women exhibited similar trends to men, while premenopausal women showed a tendency towards higher BMD, although statistical significance was not consistently achieved. Subgroup and sensitivity analyses supported the robustness of the findings.

**Conclusion:**

The study suggests a negative association between resolved HBV infection and femoral and spinal BMD in adult men in the United States. It highlights the importance of routine bone density assessments and the consideration of anti-osteoporotic therapy, if necessary, in individuals with resolved HBV infection.

## 1 Introduction

Hepatitis B virus (HBV) infection is a global health problem, affecting approximately 240 million people worldwide (1) and 2.2 million in the United States (2). In addition to its potential to cause severe liver disease and liver-related mortality, HBV infection can also lead to extrahepatic complications that significantly impact patients’ prognosis (3, 4). One such complication is osteoporosis, a systemic bone disease characterized by low bone mineral density (BMD). Osteoporosis is a prevalent health concern, with an estimated 12.3 million Americans currently affected by the condition (5). Individuals with chronic hepatitis B infection face a higher risk, with prevalence rates of osteoporosis and osteopenia at 11.5%-12.8% and 23%-24.3%, respectively, compared to lower rates of 4.1%-4.7% for osteoporosis and 9.5%-10.1% for osteopenia in healthy controls (6). Furthermore, individuals with chronic hepatitis B infection have 1.09 times higher odds of developing osteoporosis or experiencing fractures compared to matched controls (7). There is also a trend toward an increase prevalence of osteoporosis among HBV-infected individuals (8). Osteoporosis is associated with a heightened risk of fragility fractures and significantly affects patients’ quality of life. Specifically, femoral region osteoporosis is linked to an elevated risk of all-cause mortality (9).

Resolved HBV infection refers to a state in which an individual who was previously infected with HBV has cleared the virus from their bloodstream and achieved a non-infectious state. This state is typically characterized by the absence of detectable HBV surface antigen (HBsAg) and the presence of HBV core antigen antibody (HBcAb) (10, 11). It is not uncommon, and an estimated 9.87 million U.S. adults are affected (12). Resolved HBV infection is considered a functional cure of hepatitis B, leading to considerable neglect in health management. Nevertheless, individuals in this group do not appear to be fully treated as a normal condition due to the presence of covalently closed circular DNA (cccDNA) in the liver, which can cause ongoing liver damage (12) and potential viral reactivation, especially under conditions of immune suppression (10). Therefore, individuals with resolved HBV infection require special medical monitoring and regular checkups to detect any signs of liver damage and viral reactivation. They may also need antiviral therapy as a preventive measure during immune suppression to prevent viral reactivation. Consequently, there is a possibility that individuals with resolved HBV infection are at risk of developing osteoporosis, similar to those with active HBV infection. However, currently, there is a lack of research on the association between resolved HBV infection and osteoporosis.

In this cross-sectional study, we aimed to investigate the association between resolved HBV infection and osteoporosis in a large population-based study, utilizing data from the National Health and Nutrition Examination Survey (NHANES).

## 2 Methods

### 2.1 Data source and subjects

This cross-sectional study is based on data from NHANES, a significant program conducted by the National Center for Health Statistics. NHANES aims to evaluate the health and nutritional status of individuals, including both adults and children, across the United States. The survey collects a wide range of information, including demographic, dietary, examination, laboratory, and questionnaire data, from a nationally representative sample of approximately 5,000 people each year and publishes its findings biennially.

Starting from the 2005-2006 cycle, the NHANES survey began including measurements of femoral and spinal BMD, with the exception of the 2011-2012 and 2015-2016 cycles, during which femoral BMD was not assessed. As a result, this study incorporated data from five NHANES cycles: 2005-2006, 2007-2008, 2009-2010, 2013-2014, and 2017-2018.

In this cross-sectional study, we included individuals aged 20-79 years with negative HBsAg. To minimize potential age bias, we did not include individuals aged 80 years or older due to the limitation of categorizing them all as 80 years old. We excluded participants with incomplete or invalid data on femoral or spinal BMD, as well as those with missing information on covariates and menstrual history. Women were classified into two groups based on menopausal status, following previous literature: premenopausal, if they were still experiencing menstruation or had amenorrhea due to factors such as pregnancy, breastfeeding, or medical conditions/treatments during the previous 12 months; postmenopausal, if they had not had menstrual periods for at least 12 months due to natural menopause or hysterectomy (13, 14). No HBV infection was defined as testing negative for both HBsAg and HBcAg, while resolved HBV infection was defined as testing negative for HBsAg but positive for HBcAg.

The study protocol was approved by the National Center for Health Statistics Research Ethics Review Board. Written informed consent was obtained from all participants. This study followed the Strengthening the Reporting of Observational Studies in Epidemiology (STROBE) reporting guideline.

### 2.2 BMD measurement

Femoral and spinal BMD measurements were conducted using dual-energy X-ray absorptiometry (DXA) in the NHANES. For femoral BMD, Hologic QDR-4500A fan-beam densitometers (Hologic, Inc., Bedford, Massachusetts) were used in all five cycles. As for spinal BMD, Hologic QDR-4500A fan-beam densitometers were used in the 2005-2010 cycles and Hologic Discovery model A densitometers (Hologic, Inc., Bedford, Massachusetts) were used in the 2013-2014 and 2017-2018 cycles. The initial analysis of femoral and spinal BMD scans was performed using Hologic Discovery v12.4 software and then reanalyzed using Hologic APEX v3.0 software in the 2005-2008 cycles. Subsequently, the scans were analyzed with Hologic APEX v3.0 software in the 2009-2010 cycle, and with Hologic APEX v4.0 software in the 2013-2014 and 2017-2018 cycles.

### 2.3 Covariates

Based on previous literature, clinical experience, and the availability of data within NHANES, the following covariates were included in the analysis: age, race, educational level, marital status, poverty income ratio, physical activity, smoking status, body mass index (BMI), hepatitis B surface antibody (HBsAb), platelets, albumin, alanine aminotransferase (ALT), aspartate aminotransferase (AST), gamma-glutamyltransferase (GGT), total bilirubin, alkaline phosphatase, estimated glomerular filtration rate (eGFR), cholesterol, triglycerides, fasting glucose (FBG), glycosylated hemoglobin (HbA1c), uric acid, total calcium, phosphorus, history of hypertension, and history of diabetes (4, 15, 16).

According to NHANES, the ethnic groups included in the study are categorized as follows: Mexican American, other Hispanic, non-Hispanic white, non-Hispanic black, and other races. Educational level was divided into three categories: under high school, high school or equivalent, and above high school. Marital status was categorized into two groups based on whether individuals lived with a partner. The poverty income ratio was categorized into three levels: ≤1.3, 1.31 to 3.5, and >3.5 (17, 18). Physical activity was classified as either vigorous/moderate or sedentary based on responses to the questions “average level of physical activity each day” or “moderate work activity”. Participants who had smoked 100 cigarettes or more were categorized as smokers, while those who had never smoked or had smoked fewer than 100 cigarettes during their lifetime were classified as non-smokers (4, 19). BMI was calculated as body weight (kg) divided by body height squared (m^2^), and eGFR was calculated using the CKD-EPI formula. Subjects were considered to have hypertension if they answered “yes” to the questions “have been told you have high blood pressure” or “take a prescription for high blood pressure”. They were considered to have diabetes if they responded “yes” to the questions “doctor has told you have diabetes”, “take insulin now”, or “take diabetic pills to lower blood sugar”.

### 2.4 Statistical analysis

Descriptive analysis was performed on all participants, where categorical variables were presented as numbers with proportions (%), and continuous variables were reported as means with standard deviation (SD) or medians with interquartile range (IQR), depending on their distribution. To evaluate differences between groups, appropriate statistical tests were applied. The chi-squared test was used for categorical variables, one-way analysis of variance (ANOVA) was employed for normally distributed variables, and the Kruskal-Wallis test was utilized for variables with skewed distributions.

To minimize allocation bias and confounding, linear regression with propensity score matching (PSM) was employed. A 1:1 nearest neighbor matching algorithm with a caliper width of 0.3 was used. All covariates were included in creating the propensity score. The standardized mean difference (SMD) was used to assess the degree of propensity score matching, with a threshold of less than 0.1 considered acceptable. Using the estimated propensity scores as weights, the standardized mortality ratio weighting (SMRW), pairwise algorithmic (PA), and overlap weight (OW) models were used to generate a weighted cohort.

In the subgroup analysis, multivariate linear regression was utilized to evaluate the heterogeneity among subgroups. The regression models included the covariates used in the PSM algorithm for sample matching. Additionally, a likelihood ratio test was conducted to assess the interaction between subgroups and resolved HBV infection.

To assess the robustness of the findings to the matching method, additional analyses were performed using the full cohort. Multivariate linear regression analyses were conducted to examine the independent associations after adjusting for all covariates listed in **Tables 1**–**3**. The propensity score was also included as a covariate in the analysis. In addition, stricter exclusion criteria were applied. Participants with a history of chronic liver disease, chronic kidney disease, and cancer were excluded from the analysis, as well as those using steroids, anti-osteoporotic drugs, and hormone replacement therapy, and individuals who were classified as heavy drinkers. Medical history and use of osteoporosis-related medications were assessed through a questionnaire. Heavy drinking was defined as consuming more than 4 standard drinks for men or 3 drinks for women on any day, according to the National Institute on Alcohol Abuse and Alcoholism (20). Further study was conducted, excluding participants with elevated serum ALT or Fibrosis-4 (FIB-4) index > 1.45, to eliminate the potential confounding effects of active hepatitis or cirrhosis. Serum ALT can serve as an indicator of necroinflammatory activity and is a relevant marker available in the NHANES study. The FIB-4 index is a simple and widely used non-invasive tool for assessing liver fibrosis and cirrhosis. It is more precise than AST-to-platelet ratio index (APRI), another commonly used non-invasive tool for assessing liver fibrosis, in chronic hepatitis B (21). It is calculated using the formula: FIB-4 index = [Age (years) × AST (U/L)]/[Platelet count (10^9^ cells/L) × √ALT (U/L)]. The cut-off value of FIB-4 index ≤1.45 was used to differentiate moderate fibrosis from severe fibrosis (21).

**Table 1:** Baseline characteristics of men participants before and after propensity score matching.

	Unmatched Participants				Propensity Score Matched Participants			
	Total (n=5337)	No HBV infection (n=4907)	Resolved HBV infection (n=430)	SMD	Total (n=688)	No HBV infection (n=344)	Resolved HBV infection (n=344)	SMD
Age (years)	46.4 ± 15.5	45.7 ± 15.5	55.0 ± 12.4	0.665	52.3 ± 14.0	51.4 ± 15.3	53.2 ± 12.5	0.131
Race, n (%)				0.854				0.119
Mexican American	1044 (19.6)	1004 (20.5)	40 (9.3)		68 (9.9)	31 (9)	37 (10.8)	
Other Hispanic	429 (8.0)	385 (7.8)	44 (10.2)		72 (10.5)	38 (11)	34 (9.9)	
Non-Hispanic White	2461 (46.1)	2366 (48.2)	95 (22.1)		176 (25.6)	83 (24.1)	93 (27)	
Non-Hispanic Black	1032 (19.3)	883 (18)	149 (34.7)		240 (34.9)	120 (34.9)	120 (34.9)	
Other Race	371 (7.0)	269 (5.5)	102 (23.7)		132 (19.2)	72 (20.9)	60 (17.4)	
Education level, n (%)				0.068				0.089
Under high school	1438 (26.9)	1317 (26.8)	121 (28.1)		186 (27.0)	88 (25.6)	98 (28.5)	
High school or equivalent	1291 (24.2)	1179 (24)	112 (26)		165 (24.0)	80 (23.3)	85 (24.7)	
Above High school	2608 (48.9)	2411 (49.1)	197 (45.8)		337 (49.0)	176 (51.2)	161 (46.8)	
Living with a partner, n (%)	3635 (68.1)	3353 (68.3)	282 (65.6)	0.058	442 (64.2)	220 (64)	222 (64.5)	0.012
Poverty income ratio, n (%)				0.249				0.038
≤1.30	1544 (28.9)	1382 (28.2)	162 (37.7)		240 (34.9)	117 (34)	123 (35.8)	
1.31–3.50	1958 (36.7)	1798 (36.6)	160 (37.2)		260 (37.8)	131 (38.1)	129 (37.5)	
>3.50	1835 (34.4)	1727 (35.2)	108 (25.1)		188 (27.3)	96 (27.9)	92 (26.7)	
Vigorous/Moderate physical activity, n (%)	2884 (54.0)	2687 (54.8)	197 (45.8)	0.18	334 (48.5)	163 (47.4)	171 (49.7)	0.047
Smoker, n (%)	2881 (54.0)	2613 (53.3)	268 (62.3)	0.185	410 (59.6)	202 (58.7)	208 (60.5)	0.036
BMI (kg/m^2^)	27.9 ± 5.0	28.0 ± 5.0	26.6 ± 4.5	0.303	27.0 ± 4.5	27.1 ± 4.7	26.9 ± 4.3	0.05
HBsAb (+), n (%)	1062 (19.9)	740 (15.1)	322 (74.9)	1.504	481 (69.9)	244 (70.9)	237 (68.9)	0.044
Platelets (1000 cells/uL)	243.7 ± 61.1	244.5 ± 60.8	234.0 ± 63.5	0.169	235.9 ± 59.6	233.7 ± 53.0	238.2 ± 65.5	0.076
Albumin (g/L)	43.4 ± 3.1	43.5 ± 3.0	42.3 ± 3.5	0.362	42.7 ± 3.3	42.7 ± 3.2	42.6 ± 3.4	0.027
ALT (U/L)	25.0 (20.0, 34.0)	25.0 (20.0, 34.0)	24.0 (19.0, 36.0)	0.114	25.0 (19.0, 35.0)	24.5 (19.0, 34.0)	25.0 (19.8, 37.0)	0.021
AST (U/L)	25.0 (21.0, 30.0)	25.0 (21.0, 30.0)	25.0 (21.0, 33.0)	0.182	25.0 (21.0, 31.0)	25.0 (21.0, 31.0)	25.0 (21.0, 33.0)	0.018
GGT (U/L)	24.0 (18.0, 37.0)	24.0 (17.0, 36.0)	27.0 (19.0, 43.0)	0.216	26.0 (18.0, 43.0)	25.0 (18.0, 40.5)	28.0 (19.0, 44.2)	0.014
Total bilirubin (umol/L)	14.0 ± 5.8	14.1 ± 5.8	12.9 ± 5.6	0.203	13.4 ± 5.4	13.5 ± 5.4	13.3 ± 5.5	0.047
Alkaline phosphatase (U/L)	69.6 ± 24.4	69.5 ± 23.3	71.2 ± 34.3	0.059	71.7 ± 33.5	72.6 ± 30.3	70.8 ± 36.4	0.055
eGFR (ml/min/1.73m^2^)	95.9 ± 19.8	96.4 ± 19.8	90.2 ± 19.2	0.315	91.6 ± 20.4	91.9 ± 21.4	91.2 ± 19.3	0.035
Cholesterol (mmol/L)	5.1 ± 1.1	5.1 ± 1.1	5.0 ± 1.1	0.063	5.0 ± 1.1	5.0 ± 1.1	5.0 ± 1.0	0.034
Triglycerides (mmol/L)	1.5 (1.0, 2.4)	1.5 (1.0, 2.4)	1.4 (1.0, 2.3)	0.093	1.3 (0.9, 2.2)	1.2 (0.9, 2.0)	1.4 (1.0, 2.3)	0.005
FBG (mmol/L)	5.7 ± 2.1	5.7 ± 2.1	6.0 ± 2.4	0.135	5.9 ± 2.5	5.8 ± 2.4	6.0 ± 2.6	0.081
HBA1c (%)	5.7 ± 1.0	5.7 ± 1.0	5.9 ± 1.3	0.197	5.8 ± 1.3	5.8 ± 1.2	5.9 ± 1.4	0.006
Uric acid (umol/L)	357.2 ± 74.8	357.4 ± 74.1	354.2 ± 82.3	0.041	356.5 ± 78.5	357.7 ± 74.2	355.2 ± 82.7	0.031
Total calcium (mmol/L)	2.4 ± 0.1	2.4 ± 0.1	2.4 ± 0.1	0.078	2.4 ± 0.1	2.4 ± 0.1	2.4 ± 0.1	0.008
Phosphorus (mmol/L)	1.2 ± 0.2	1.2 ± 0.2	1.2 ± 0.2	0.015	1.2 ± 0.2	1.2 ± 0.2	1.2 ± 0.2	0.048
Hypertension, n (%)	1939 (36.3)	1749 (35.6)	190 (44.2)	0.175	289 (42.0)	148 (43)	141 (41)	0.041
Diabetes, n (%)	793 (14.9)	696 (14.2)	97 (22.6)	0.218	134 (19.5)	66 (19.2)	68 (19.8)	0.015

HBcAb, hepatitis B surface core antibody; HBsAb, hepatitis B surface; BMI, body mass index; ALT, alanine transaminase; AST, aspartate transaminase; GGT, gamma-glutamyl transpeptidase; eGFR, estimated glomerular filtration rate; FBG, fasting blood-glucose; HBA1c, glycosylated hemoglobin; BMD, bone mineral density.

**Table 2:** Baseline characteristics of postmenopausal women participants before and after propensity score matching.

	Unmatched Participants				Propensity Score Matched Participants			
	Total (n=2238)	No HBV infection (n=2030)	Resolved HBV infection (n=208)	SMD	Total (n=304)	No HBV infection (n=152)	Resolved HBV infection (n=152)	SMD
Age (years)	60.2 ± 9.5	60.1 ± 9.6	61.4 ± 8.0	0.149	60.0 ± 8.7	59.8 ± 9.1	60.3 ± 8.3	0.063
Race, n (%)				1.149				0.199
Mexican American	368 (16.4)	349 (17.2)	19 (9.1)		35 (11.5)	17 (11.2)	18 (11.8)	
Other Hispanic	195 (8.7)	166 (8.2)	29 (13.9)		42 (13.8)	16 (10.5)	26 (17.1)	
Non-Hispanic White	1092 (48.8)	1063 (52.4)	29 (13.9)		60 (19.7)	31 (20.4)	29 (19.1)	
Non-Hispanic Black	435 (19.4)	365 (18)	70 (33.7)		111 (36.5)	58 (38.2)	53 (34.9)	
Other Race	148 (6.6)	87 (4.3)	61 (29.3)		56 (18.4)	30 (19.7)	26 (17.1)	
Education level, n (%)				0.19				0.196
Under high school	580 (25.9)	510 (25.1)	70 (33.7)		102 (33.6)	44 (28.9)	58 (38.2)	
High school or equivalent	552 (24.7)	508 (25)	44 (21.2)		65 (21.4)	35 (23)	30 (19.7)	
Above High school	1106 (49.4)	1012 (49.9)	94 (45.2)		137 (45.1)	73 (48)	64 (42.1)	
Living with a partner, n (%)	1257 (56.2)	1167 (57.5)	90 (43.3)	0.287	141 (46.4)	72 (47.4)	69 (45.4)	0.04
Poverty income ratio, n (%)				0.204				0.192
≤1.30	629 (28.1)	555 (27.3)	74 (35.6)		99 (32.6)	43 (28.3)	56 (36.8)	
1.31–3.50	836 (37.4)	759 (37.4)	77 (37)		118 (38.8)	61 (40.1)	57 (37.5)	
>3.50	773 (34.5)	716 (35.3)	57 (27.4)		87 (28.6)	48 (31.6)	39 (25.7)	
Vigorous/Moderate physical activity, n (%)	922 (41.2)	850 (41.9)	72 (34.6)	0.15	110 (36.2)	55 (36.2)	55 (36.2)	<0.001
Smoker, n (%)	955 (42.7)	881 (43.4)	74 (35.6)	0.161	118 (38.8)	58 (38.2)	60 (39.5)	0.027
BMI (kg/m^2^)	28.6 ± 6.1	28.7 ± 6.1	28.2 ± 5.9	0.083	28.5 ± 6.0	28.4 ± 6.1	28.6 ± 5.9	0.041
HBsAb (+), n (%)	420 (18.8)	255 (12.6)	165 (79.3)	1.804	223 (73.4)	114 (75)	109 (71.7)	0.074
Platelets (1000 cells/uL)	264.1 ± 66.6	264.7 ± 66.5	258.2 ± 67.6	0.097	263.4 ± 65.4	260.8 ± 62.1	266.0 ± 68.7	0.08
Albumin (g/L)	41.9 ± 2.9	42.0 ± 2.9	41.5 ± 2.8	0.167	41.6 ± 2.7	41.7 ± 2.7	41.4 ± 2.7	0.128
ALT (U/L)	20.0 (16.0, 25.0)	20.0 (16.0, 25.0)	19.0 (15.0, 24.2)	0.038	19.0 (16.0, 25.0)	20.0 (16.0, 26.2)	19.0 (15.8, 24.0)	0.001
AST (U/L)	23.0 (20.0, 27.0)	23.0 (20.0, 27.0)	22.0 (19.8, 26.0)	0.053	23.0 (19.0, 26.0)	22.5 (19.0, 27.0)	23.0 (20.0, 26.0)	0.015
GGT (U/L)	19.0 (14.0, 29.0)	19.0 (14.0, 29.0)	20.0 (15.0, 30.0)	0.11	19.0 (15.0, 32.0)	19.0 (15.0, 31.2)	19.5 (15.0, 32.0)	0.031
Total bilirubin (umol/L)	11.1 ± 3.9	11.2 ± 3.9	10.1 ± 4.0	0.303	10.5 ± 3.8	10.4 ± 3.7	10.5 ± 4.0	0.026
Alkaline phosphatase (U/L)	75.3 ± 25.4	74.9 ± 25.0	78.8 ± 28.9	0.141	77.4 ± 25.1	76.5 ± 27.0	78.4 ± 23.2	0.077
eGFR (ml/min/1.73m^2^)	84.8 ± 18.2	84.8 ± 18.3	85.1 ± 17.9	0.02	85.7 ± 17.8	85.6 ± 18.0	85.7 ± 17.6	0.004
Cholesterol (mmol/L)	5.4 ± 1.1	5.4 ± 1.1	5.4 ± 1.0	0.011	5.4 ± 1.1	5.5 ± 1.2	5.4 ± 1.0	0.069
Triglycerides (mmol/L)	1.5 (1.0, 2.2)	1.5 (1.0, 2.2)	1.3 (0.9, 2.2)	0.091	1.4 (0.9, 2.3)	1.4 (0.9, 2.3)	1.4 (0.9, 2.3)	0.066
FBG (mmol/L)	5.8 ± 2.3	5.8 ± 2.2	5.9 ± 2.3	0.021	5.9 ± 2.2	6.0 ± 2.0	5.8 ± 2.4	0.076
HBA1c (%)	5.9 ± 1.1	5.9 ± 1.1	6.0 ± 1.2	0.131	6.0 ± 1.2	6.1 ± 1.1	6.0 ± 1.3	0.052
Uric acid (umol/L)	300.0 ± 75.2	299.4 ± 75.5	306.0 ± 73.0	0.088	304.1 ± 77.6	301.8 ± 76.8	306.4 ± 78.7	0.058
Total calcium (mmol/L)	2.4 ± 0.1	2.4 ± 0.1	2.4 ± 0.1	0.031	2.4 ± 0.1	2.4 ± 0.1	2.4 ± 0.1	0.029
Phosphorus (mmol/L)	1.3 ± 0.2	1.3 ± 0.2	1.2 ± 0.2	0.152	1.2 ± 0.2	1.2 ± 0.1	1.2 ± 0.2	0.076
Hypertension, n (%)	1288 (57.6)	1150 (56.7)	138 (66.3)	0.2	191 (62.8)	98 (64.5)	93 (61.2)	0.068
Diabetes, n (%)	494 (22.1)	443 (21.8)	51 (24.5)	0.064	82 (27.0)	43 (28.3)	39 (25.7)	0.059

HBcAb, hepatitis B surface core antibody; HBsAb, hepatitis B surface; BMI, body mass index; ALT, alanine transaminase; AST, aspartate transaminase; GGT, gamma-glutamyl transpeptidase; eGFR, estimated glomerular filtration rate; FBG, fasting blood-glucose; HBA1c, glycosylated hemoglobin; BMD, bone mineral density.

**Table 3:** Baseline characteristics of premenopausal women participants before and after propensity score matching.

	Unmatched Participants				Propensity Score Matched Participants			
	Total (n=2758)	No HBV infection (n=2659)	Resolved HBV infection (n=99)	SMD	Total (n=174)	No HBV infection (n=87)	Resolved HBV infection (n=87)	SMD
Age (years)	36.4 ± 9.4	36.3 ± 9.4	40.2 ± 9.0	0.427	39.1 ± 9.4	39.0 ± 9.8	39.1 ± 9.0	0.021
Race, n (%)				1.187				0.182
Mexican American	559 (20.3)	553 (20.8)	6 (6.1)		12 (6.9)	6 (6.9)	6 (6.9)	
Other Hispanic	278 (10.1)	267 (10)	11 (11.1)		21 (12.1)	10 (11.5)	11 (12.6)	
Non-Hispanic White	1242 (45.0)	1229 (46.2)	13 (13.1)		25 (14.4)	12 (13.8)	13 (14.9)	
Non-Hispanic Black	498 (18.1)	459 (17.3)	39 (39.4)		65 (37.4)	30 (34.5)	35 (40.2)	
Other Race	181 (6.6)	151 (5.7)	30 (30.3)		51 (29.3)	29 (33.3)	22 (25.3)	
Education level, n (%)				0.259				0.137
Under high school	598 (21.7)	576 (21.7)	22 (22.2)		33 (19.0)	15 (17.2)	18 (20.7)	
High school or equivalent	534 (19.4)	505 (19)	29 (29.3)		51 (29.3)	28 (32.2)	23 (26.4)	
Above High school	1626 (59.0)	1578 (59.3)	48 (48.5)		90 (51.7)	44 (50.6)	46 (52.9)	
Living with a partner, n (%)	1687 (61.2)	1626 (61.2)	61 (61.6)	0.01	101 (58.0)	50 (57.5)	51 (58.6)	0.023
Poverty income ratio, n (%)				0.247				0.091
≤1.30	879 (31.9)	846 (31.8)	33 (33.3)		58 (33.3)	29 (33.3)	29 (33.3)	
1.31–3.50	989 (35.9)	945 (35.5)	44 (44.4)		79 (45.4)	41 (47.1)	38 (43.7)	
>3.50	890 (32.3)	868 (32.6)	22 (22.2)		37 (21.3)	17 (19.5)	20 (23)	
Vigorous/Moderate physical activity, n (%)	1296 (47.0)	1256 (47.2)	40 (40.4)	0.138	82 (47.1)	43 (49.4)	39 (44.8)	0.092
Smoker, n (%)	983 (35.6)	947 (35.6)	36 (36.4)	0.016	62 (35.6)	29 (33.3)	33 (37.9)	0.096
BMI (kg/m^2^)	27.9 ± 6.4	27.9 ± 6.4	27.0 ± 7.3	0.137	27.3 ± 6.9	27.1 ± 6.2	27.5 ± 7.6	0.052
HBsAb (+), n (%)	823 (29.8)	740 (27.8)	83 (83.8)	1.366	140 (80.5)	69 (79.3)	71 (81.6)	0.058
Platelets (1000 cells/uL)	281.9 ± 72.6	282.0 ± 72.5	277.3 ± 75.3	0.064	280.2 ± 73.4	278.0 ± 72.8	282.5 ± 74.4	0.062
Albumin (g/L)	41.7 ± 3.1	41.7 ± 3.1	41.1 ± 3.3	0.206	41.2 ± 2.9	41.4 ± 2.6	41.1 ± 3.1	0.104
ALT (U/L)	17.0 (14.0, 23.0)	17.0 (14.0, 22.0)	18.0 (15.0, 25.0)	0.104	18.0 (14.2, 23.0)	17.0 (14.0, 21.0)	19.0 (15.0, 26.0)	0.012
AST (U/L)	21.0 (18.0, 24.0)	21.0 (18.0, 24.0)	22.0 (20.0, 25.5)	0.119	21.0 (19.0, 25.0)	20.0 (18.0, 23.0)	22.0 (20.0, 26.0)	0.07
GGT (U/L)	16.0 (12.0, 22.0)	15.0 (12.0, 22.0)	17.0 (13.0, 27.5)	0.141	17.0 (12.0, 26.0)	16.0 (12.0, 25.0)	17.0 (13.0, 28.0)	0.076
Total bilirubin (umol/L)	11.1 ± 6.0	11.1 ± 6.1	10.0 ± 3.7	0.225	10.2 ± 4.3	10.2 ± 4.7	10.2 ± 3.8	0.005
Alkaline phosphatase (U/L)	62.2 ± 20.4	62.3 ± 20.5	60.3 ± 19.0	0.103	61.7 ± 19.8	62.5 ± 20.1	60.9 ± 19.5	0.077
eGFR (ml/min/1.73m^2^)	106.1 ± 16.6	106.2 ± 16.7	103.4 ± 15.2	0.172	104.6 ± 15.7	105.8 ± 15.9	103.5 ± 15.5	0.147
Cholesterol (mmol/L)	5.0 ± 1.0	5.0 ± 1.0	4.8 ± 1.0	0.164	4.8 ± 0.9	4.8 ± 0.8	4.9 ± 1.0	0.106
Triglycerides (mmol/L)	1.1 (0.7, 1.6)	1.1 (0.8, 1.6)	1.1 (0.7, 1.7)	0.055	1.0 (0.7, 1.6)	1.0 (0.7, 1.4)	1.1 (0.7, 1.7)	0.065
FBG (mmol/L)	5.1 ± 1.7	5.1 ± 1.6	5.7 ± 2.4	0.287	5.4 ± 2.1	5.3 ± 1.9	5.6 ± 2.3	0.134
HBA1c (%)	5.4 ± 0.8	5.4 ± 0.8	5.7 ± 1.2	0.292	5.6 ± 1.1	5.5 ± 0.9	5.6 ± 1.2	0.096
Uric acid (umol/L)	265.9 ± 60.8	266.0 ± 60.7	264.3 ± 65.2	0.027	265.4 ± 61.4	267.8 ± 55.8	263.0 ± 66.9	0.078
Total calcium (mmol/L)	2.3 ± 0.1	2.3 ± 0.1	2.3 ± 0.1	0.012	2.3 ± 0.1	2.3 ± 0.1	2.3 ± 0.1	0.037
Phosphorus (mmol/L)	1.2 ± 0.2	1.2 ± 0.2	1.2 ± 0.2	0.055	1.2 ± 0.2	1.2 ± 0.2	1.2 ± 0.2	0.039
Hypertension, n (%)	490 (17.8)	464 (17.5)	26 (26.3)	0.214	42 (24.1)	19 (21.8)	23 (26.4)	0.108
Diabetes, n (%)	170 (6.2)	156 (5.9)	14 (14.1)	0.278	19 (10.9)	8 (9.2)	11 (12.6)	0.111

HBcAb, hepatitis B surface core antibody; HBsAb, hepatitis B surface; BMI, body mass index; ALT, alanine transaminase; AST, aspartate transaminase; GGT, gamma-glutamyl transpeptidase; eGFR, estimated glomerular filtration rate; FBG, fasting blood-glucose; HBA1c, glycosylated hemoglobin; BMD, bone mineral density.

All the analyses were performed with the statistical software packages R (http://www.R-project.org, The R Foundation) and Free Statistics software version 1.7. By a two-tailed testing, a p-value of <0.05 was declared significant.

## 3 Results

### 3.1 Study population

**Figure 1:**
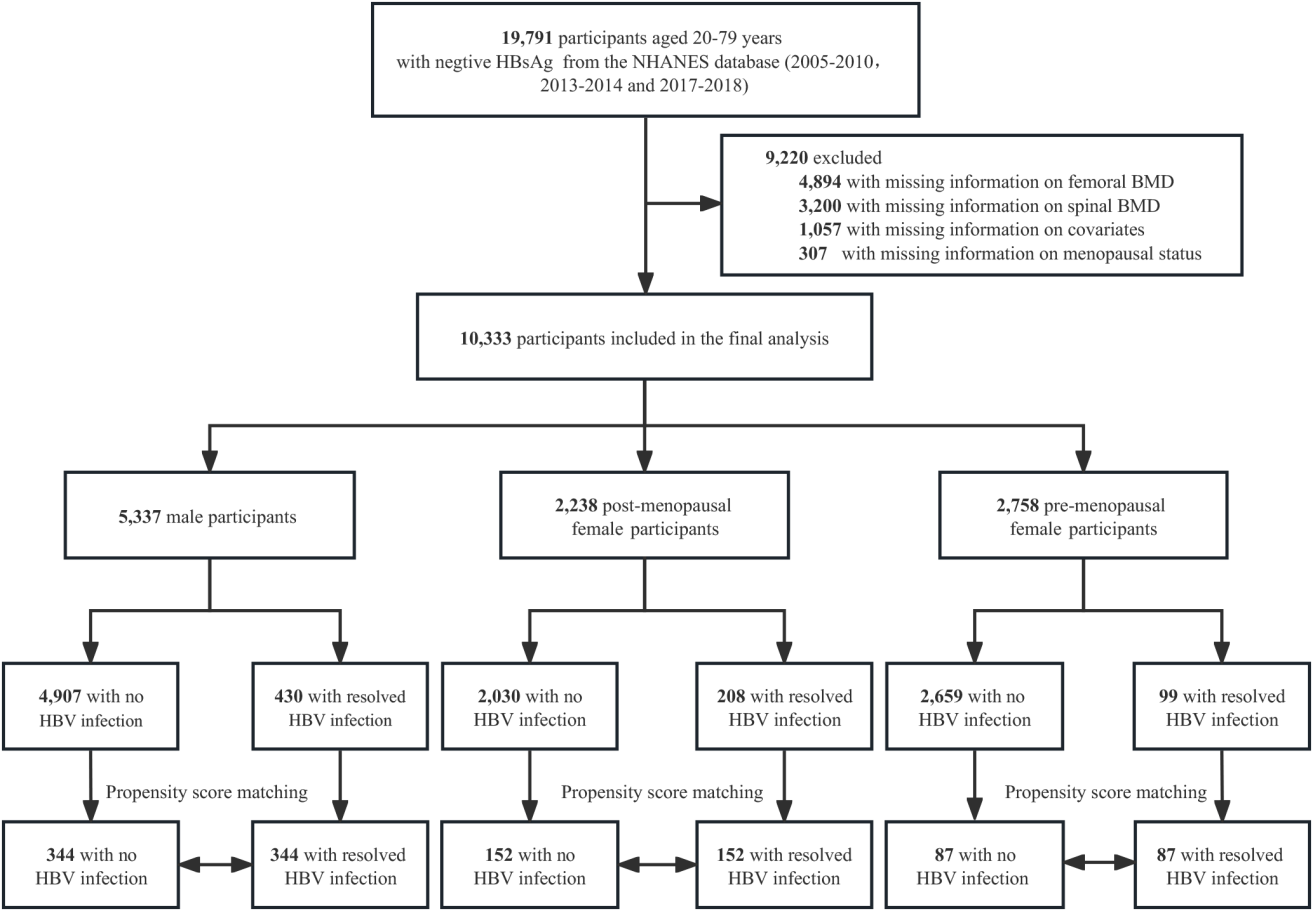
Flowchart of study participant enrollment.

A total of 19,791 individuals aged 20-79 years with negative HBsAg from the NHANES cycles of 2005-2010, 2013-2014, and 2017-2018 were initially included in this cross-sectional study. After excluding individuals with missing data on femoral BMD (n=4,894), spinal BMD (n=3,200), covariates (n=1,057), and menstrual history (n=307), a final sample of 10,333 participants were included in the analysis. This final sample consisted of 5,337 men, 2,238 postmenopausal women, and 2,758 premenopausal women. Following PSM, 344, 152, and 87 pairs were matched in men, postmenopausal women, and premenopausal women, respectively. The enrollment flowchart is presented in **Figure 1**.

### 3.2 Baseline characteristics

Out of the 10,333 participants, 737 (7.1%) were found to have resolved HBV infection. This included 430 (8.1%) men, 208 (9.3%) postmenopausal women, and 99 (3.6%) premenopausal women.

After PSM, participant characteristics were well-balanced between the no HBV infection and resolved HBV infection groups. In men, postmenopausal women, and premenopausal women, the mean age was 52.3 ± 14.0 years, 60.0 ± 8.7 years, and 39.1 ± 9.4 years, respectively; the mean BMI was 27.0 ± 4.5 kg/m^2^, 28.5 ± 6.0 kg/m^2^, and 27.3 ± 6.9 kg/m^2^, respectively; and the HBsAb positive rate was 69.9%, 73.4%, and 80.5%, respectively. Detailed demographic characteristics of all participants and the matched cohort are presented in **Tables 1** to **3**.

### 3.3 Association of resolved HBV infection with femoral and spinal BMD

Before PSM, men with resolved HBV infection showed significantly lower femoral and spinal BMD compared to those with no HBV infection (0.828 ± 0.144 g/cm^2^ vs. 0.878 ± 0.146 g/cm^2^, p<0.001 and 1.027 ± 0.153 g/cm^2^ vs. 1.057 ± 0.143 g/cm^2^, p<0.001, respectively). After PSM, similar findings were still observed (0.840 ± 0.145 g/cm^2^ vs. 0.865 ± 0.155 g/cm^2^, p=0.038 and 1.038 ± 0.152 g/cm^2^ vs. 1.063 ± 0.159 g/cm^2^, p=0.018, respectively). In postmenopausal women, similar to men, the resolved HBV infection group exhibited lower femoral and spinal BMD both before and after PSM compared to the no HBV infection group. In premenopausal women, the trend was the opposite, with higher BMD observed in the resolved HBV infection group. However, these differences were not statistically significant (**Table 4**).

**Table 4:** Femoral and spinal BMD (g/cm^2^) before and after propensity score matching.

	Unmatched Participants				Propensity Score Matched Participants			
	Total	No HBV infection	Resolved HBV infection	P-value	Total	No HBV infection	Resolved HBV infection	P-value
Men	n = 5337	n = 4907	n = 430		n = 688	n = 344	n = 344	
Femoral BMD	0.874 ± 0.147	0.878 ± 0.146	0.828 ± 0.144	< 0.001	0.853 ± 0.151	0.865 ± 0.155	0.840 ± 0.145	0.038
Spinal BMD	1.055 ± 0.144	1.057 ± 0.143	1.027 ± 0.153	< 0.001	1.050 ± 0.156	1.063 ± 0.159	1.038 ± 0.152	0.018
Postmenopausal women	n = 2238	n = 2030	n = 208		n = 304	n = 152	n = 152	
Femoral BMD	0.742 ± 0.134	0.743 ± 0.134	0.735 ± 0.140	0.417	0.751 ± 0.151	0.755 ± 0.159	0.748 ± 0.142	0.656
Spinal BMD	0.950 ± 0.157	0.951 ± 0.156	0.938 ± 0.162	0.23	0.946 ± 0.156	0.948 ± 0.161	0.943 ± 0.152	0.782
Premenopausal women	n = 2758	n = 2659	n = 99		n = 174	n = 87	n = 87	
Femoral BMD	0.862 ± 0.129	0.861 ± 0.128	0.875 ± 0.135	0.295	0.868 ± 0.131	0.853 ± 0.129	0.884 ± 0.132	0.115
Spinal BMD	1.062 ± 0.125	1.061 ± 0.125	1.075 ± 0.133	0.288	1.062 ± 0.128	1.049 ± 0.130	1.076 ± 0.125	0.162

**Figure 2:**
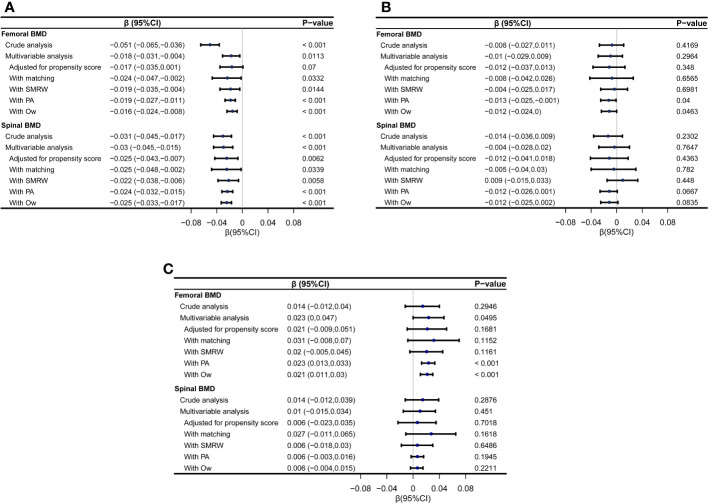
Forest plot showing the associations between resolved HBV infection and femoral and spinal BMD (g/cm^2^) using different statistical models. **(A)** the associations in men; **(B)** the associations in postmenopausal women; **(C)** the associations in premenopausal women. SMRW, the standardized mortality ratio weighting; PA, pairwise algorithmic; Ow, overlap weight.

In men, after propensity score matching, a negative association was observed between resolved HBV infection and both femoral BMD (β=-0.024, 95%CI: -0.047 to -0.002, p=0.0332) and spinal BMD (β=-0.025 95%CI: -0.048 to -0.002, p=0.0339). Consistent results were obtained using weighting analysis with SMRW, PA, and OW. In postmenopausal women, there were trends suggesting a potential negative effect of resolved HBV infection on femoral and spinal BMD, although not all reached statistical significance. Conversely, in premenopausal women, a positive association between resolved HBV infection and femoral and spinal BMD was observed, reaching statistical significance in certain models (**Figure 2**).

### 3.4 Subgroup analyses

**Figure 3:**
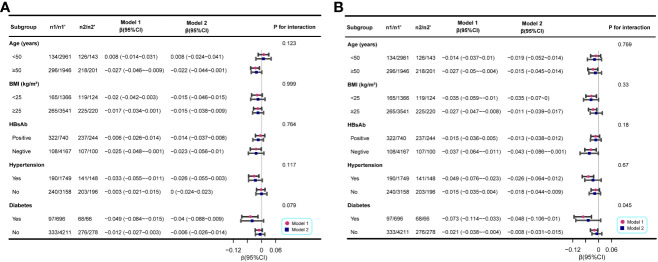
Associations between resolved HBV infection and femoral and spinal BMD (g/cm^2^) in different stratifications. **(A)** the associations between resolved HBV infection and femoral BMD; **(B)** the associations between resolved HBV infection and spinal BMD. The analysis was performed separately for the entire population and the matched population generated through PSM. n1, the number of participants with resolved HBV infection in the entire population; n1’, the number of participants with no HBV infection in the entire population; n2, the number of participants with resolved HBV infection in the matched population; n2’, the number of participants with no HBV infection in the matched population. Each stratification was adjusted for all other variables listed in **Tables 1** to **3**, except the specific stratification factor itself.

The subgroup analyses in men consistently demonstrated a negative association between resolved HBV infection and femoral and spinal BMD, irrespective of the subgroup being considered. This association was particularly notable in individuals with negative HBsAb and those with coexisting hypertension or diabetes (**Figure 3**).

### 3.5 Sensitivity analyses

Upon adjusting for all covariates listed in **Tables 1** to **3**, multivariate linear regression analyses in the full-participant cohort yielded consistent findings. In men, resolved HBV infection demonstrated a negative association with femoral BMD (β=-0.018, 95% CI: -0.031 to -0.004, p=0.0113) and spinal BMD (β=-0.03, 95% CI: -0.045 to -0.015, p<0.001). This trend persisted in postmenopausal women, although statistical significance was not reached. In contrast, in premenopausal women, a positive association was observed between resolved HBV infection and femoral BMD (β=0.023, 95% CI: 0 to 0.047, p=0.0495), while no significant association was found with spinal BMD. After adjusting for the propensity score, the results remained stable, although statistical significance was not reached in some models (**Figure 2**).

The robustness of the results was maintained when applying stricter exclusion criteria. After propensity score matching, the β values for femoral BMD and spinal BMD were as follows: for men, -0.028 (95% CI: -0.055 to -0.002, p=0.0384) and -0.033 (95% CI: -0.061 to -0.006, p=0.0184), respectively; for postmenopausal women, -0.004 (95% CI: -0.059 to 0.05, p=0.8735) and -0.016 (95% CI: -0.072 to 0.041, p=0.5805), respectively; and for premenopausal women, 0.049 (95% CI: 0.003 to 0.096, p=0.0359) and 0.015 (95% CI: -0.03 to 0.059, p=0.5068), respectively. After excluding participants with elevated serum ALT or FIB-4 index > 1.45, the results remained robust, as demonstrated in **Supplementary Table 1**.

## 4 Discussion

In this cross-sectional study utilizing a propensity score-matched design involving US adults, the findings indicated a negative association between resolved HBV infection and femoral and spinal BMD in men. The impact of resolved HBV infection on BMD in women was influenced by menopausal status, with postmenopausal women demonstrating similar trends to men. However, premenopausal women showed a tendency towards higher BMD, although statistical significance was not consistently achieved. Subgroup analyses in men consistently supported these results and further revealed that the association was more pronounced in individuals with negative HBsAb and those with coexisting hypertension or diabetes. These results remained robust when the full cohort of participants was analyzed using different adjustment models or when different exclusion criteria were applied. To the best of our knowledge, this is the first study to specifically investigate the relationship between resolved HBV infection and BMD.

Despite the substantial population affected by HBV infection, only a limited number of studies have explored the relationship between HBV infection and BMD. Analyzing data from the NHANES database, which did not differentiate between patients with and without cirrhosis, serologic HBsAg was found to be positively associated with an increased risk of reduced bone mass. The β value for femoral BMD was -0.018 (95% CI: -0.026 to -0.009, P < 0.01), and for spinal BMD was -0.020 (95% CI: -0.030 to -0.010, p < 0.01) (22). Only a few studies have specifically focused on non-cirrhotic HBV infection, which can help elucidate the direct effects of hepatitis B infection itself on BMD, excluding the influence of cirrhosis, a known high-risk factor for osteoporosis (23, 24). One study found that non-cirrhotic chronic hepatitis B infection did not pose a risk for osteoporosis or low BMD. However, this study was limited by its measurement of BMD in the phalanges and the small number of patients (n=15) (25). Other studies have reported contradictory findings. An early German study with a small sample size of non-cirrhotic HBV patients reported lower femoral and spinal BMD (26). This conclusion was supported by two subsequent large-scale clinical studies: one conducted in Korea with 11,306 participants, demonstrating a significant association between HBsAg seropositivity and lower BMD (4), and the other conducted in Taiwan with 51,144 subjects, showing an inverse association between HBV infection and BMD after adjusting for confounding factors (15). In both studies, the negative association between HBV infection and BMD was more pronounced in men, while in women, it did not reach statistical significance and even showed a positive trend with BMD in premenopausal women (4, 15). In our study involving participants with resolved HBV infection, a milder form of hepatitis B infection, the results align with those of previous studies, suggesting that even the short-term presence of hepatitis B virus or persistent cccDNA in men may have an adverse effect on bone mineral density.

The exact mechanism underlying the association between hepatitis B infection and reduced BMD is not yet fully understood. One possible cause is the activation of the immune response and the release of cytokines associated with viral hepatitis, such as interleukin-6, interleukin-1β, and tumor necrosis factor-alpha. These cytokines can interfere with the nuclear factor kappa ligand-nuclear factor kappa-osteoprotegerin (RANKL-RANK-OPG) system, which plays a crucial role in regulating bone homeostasis (24). The medication tenofovir disoproxil fumarate, commonly used to treat hepatitis B, may also directly or indirectly affect BMD (24). In some individuals with advanced liver disease, impaired liver function can lead to decreased hydroxylation of vitamin D3 to its active form (D25), resulting in elevated levels of parathyroid hormone. This hormonal imbalance can increase bone turnover and contribute to bone loss (27). Additionally, impaired liver-produced insulin-like growth factor 1, which is important for bone health, may inhibit osteoblast differentiation and proliferation, leading to bone mass loss (28). Other potential mechanisms that have been suggested including alterations in the gut microbiota and the presence of sarcopenia, a condition characterized by loss of skeletal muscle mass and strength, which can indirectly affect bone health (24).

The inconsistent results across gender and menopausal status may be attributed to the important role of estrogen in osteoprotective effects (29). This is supported by similar trends observed in studies on TSH suppression therapy after surgery for differentiated thyroid cancer, highlighting the protective properties of estrogen on bone density (30). In addition, liver damage caused by HBV infection has the potential to interfere with estrogen inactivation, resulting in elevated estrogen levels, especially in premenopausal women who naturally have higher estrogen levels, and a consequent increase in the osteoprotective effects of estrogen. Moreover, younger patients, who receive advanced health education, tend to adopt healthier lifestyles, contributing to higher bone density. Furthermore, the shorter duration of current HBV infection and cccDNA in premenopausal women, due to advances in medical treatments and their younger age, has a relatively minor impact on bone metabolism. Further research is needed to confirm these hypotheses.

HBsAb seroconversion indicates stable viral control, and its positivity is associated with a higher likelihood of sustained HBsAg loss and a lower occurrence of occult HBV/HBV reactivation (12).In our study, we observed that participants who tested positive for HBsAb had less BMD loss compared to those who tested negative for HBsAb, suggesting a protective effect of HBsAb on BMD. Previous studies have reported an association between hypertension, diabetes, and increased bone loss (31–33). In our subgroup analyses, we observed a more significant decrease in BMD among participants with comorbidities of hypertension or diabetes. These findings emphasize the importance of paying special attention to these high-risk groups.

This study has several limitations. Firstly, due to the inherent limitations of cross-sectional studies, a causal relationship between resolved HBV infection and decreased BMD cannot be established. Future well-designed cohort studies are needed to confirm this relationship. Secondly, like all retrospective analyses, there is a potential for residual confounding. We made efforts to adjust for various potential confounders and achieved a good balance in the propensity score-matched cohorts. Additionally, we conducted stratified and sensitivity analyses to minimize this bias. Thirdly, the presence of occult hepatitis B infection (OBI) could not be definitively excluded. OBI is characterized by the presence of replication-competent HBV DNA in the liver, with or without detectable HBV DNA in the blood, in individuals who test negative for serum HBsAg using current assays (34). Unfortunately, assessing HBV DNA in the liver is challenging in large-scale epidemiological studies, and data on both liver and blood HBV DNA levels were not available in NHANES. However, the combination of negative HBsAg and positive HBcAb has been widely used as a reliable surrogate marker for resolved HBV infection (35), demonstrating a specificity of over 95% and an overall accuracy of 90% in previous studies (36). In addition, it is worth noting that the duration of active HBV infection before the loss of HBs antigen could potentially influence bone demineralization. However, due to the unavailability of relevant data in the NHANES database, we were unable to include this analysis in our study. Future research should focus on investigating this aspect.

## 5 Conclusions

The findings of the cross-sectional study reveal a significant negative association between resolved HBV infection and BMD in the femoral and spinal regions among adult men in the United States. This discovery is of particular importance due to the tendency to overlook resolved HBV infection. Consequently, it is crucial to underscore the significance of routine bone density assessments and, if necessary, consider implementing anti-osteoporotic therapy in individuals with resolved HBV infection.

## Data availability statement

Publicly available datasets were analyzed in this study. This data can be found here: https://www.cdc.gov/nchs/nhanes/.

## Ethics statement

The studies involving humans were approved by The National Center for Health Statistics Research Ethics Review Board. The studies were conducted in accordance with the local legislation and institutional requirements. The participants provided their written informed consent to participate in this study.

## Author contributions

YY, ZQ, and YF conceived and designed the study. XF, QW, and XW collected the data. YY, JZ, and TZ analysed the data and drafted the manuscript. JW assisted in data analysis and proofread the manuscript. All authors contributed to the article and approved the submitted version.
