# Supplementary material for: Association between resolved hepatitis B virus infection and femoral and spinal bone mineral density in American adults: a cross-sectional study
Source: Front Endocrinol (Lausanne). 2023 Sep 27;14:1237618. doi: 10.3389/fendo.2023.1237618 (PMC10565481; doi:10.3389/fendo.2023.1237618)
Supplement: Supplementary file 1 [file DataSheet_1.docx]

Supplementary Material

Association between Resolved Hepatitis B Virus Infection and Femoral and Spinal Bone Mineral Density in American Adults: A Cross-sectional Study

Yan Yang^1†^, Jing Zeng^1†^, Tingting Zhang^1†^, Jinjing Wang^1^, Xiaojing Fan^1^, Qiaomin Wang^1^, Xuan Wang^1^, Zhengrong Qi^2*^, Yi Fang^1*^

*** Correspondence:** Zhengrong Qi: [qizhengrong@ccmu.edu.cn](mailto:qizhengrong@ccmu.edu.cn); Yi Fang: [fangyi5zhongxin@163.com](mailto:fangyi5zhongxin@163.com)

# Supplementary Tables

**Supplementary Table 1** **The associations between resolved HBV infection and femoral and spinal BMD (g/cm^2^).**

|  | **Men** | | | **Postmenopausal women** | | | **Premenopausal women** | | |
| --- | --- | --- | --- | --- | --- | --- | --- | --- | --- |
|  | **n** | **β (95%CI)** | **P-value** | **n** | **β (95%CI)** | **P-alue** | **n** | **β (95%CI)** | **P-value** |
| **Femoral BMD** |  |  |  |  |  |  |  |  |  |
| All participants | 688 | -0.024 (-0.047~-0.002) | 0.0332 | 304 | -0.008 (-0.042,0.026) | 0.6565 | 174 | 0.031 (-0.008,0.07) | 0.1152 |
| Exclusion participants with elevated ALT | 555 | -0.02 (-0.046~0.005) | 0.1122 | 262 | 0 (-0.037~0.037) | 0.9953 | 152 | 0.028 (-0.014~0.07) | 0.1972 |
| Exclusion participants with FIB-4 index >1.45 | 483 | -0.027 (-0.054~0) | 0.0503 | 211 | -0.009 (-0.05~0.031) | 0.6513 | 172 | 0.033 (-0.006~0.072) | 0.1033 |
| **Spinal BMD** |  |  |  |  |  |  |  |  |  |
| All participants | 688 | -0.025 (-0.048~-0.002) | 0.0339 | 304 | -0.005 (-0.04,0.03) | 0.782 | 174 | 0.027 (-0.011,0.065) | 0.1618 |
| Exclusion participants with elevated ALT | 555 | -0.024 (-0.05~0.003) | 0.077 | 262 | 0 (-0.039~0.039) | 0.9901 | 152 | 0.028 (-0.012~0.069) | 0.176 |
| Exclusion participants with FIB-4 index >1.45 | 483 | -0.027 (-0.053~0) | 0.0494 | 211 | -0.016 (-0.058~0.026) | 0.4537 | 172 | 0.028 (-0.011~0.066) | 0.1597 |

ALT, alanine aminotransferase; fibrosis-4 index, FIB-4 index

FIB-4 index = [Age (years) × AST (U/L)]/ [Platelet count (10^9^ cells/L) × √ALT (U/L)]

# Supplementary Figures


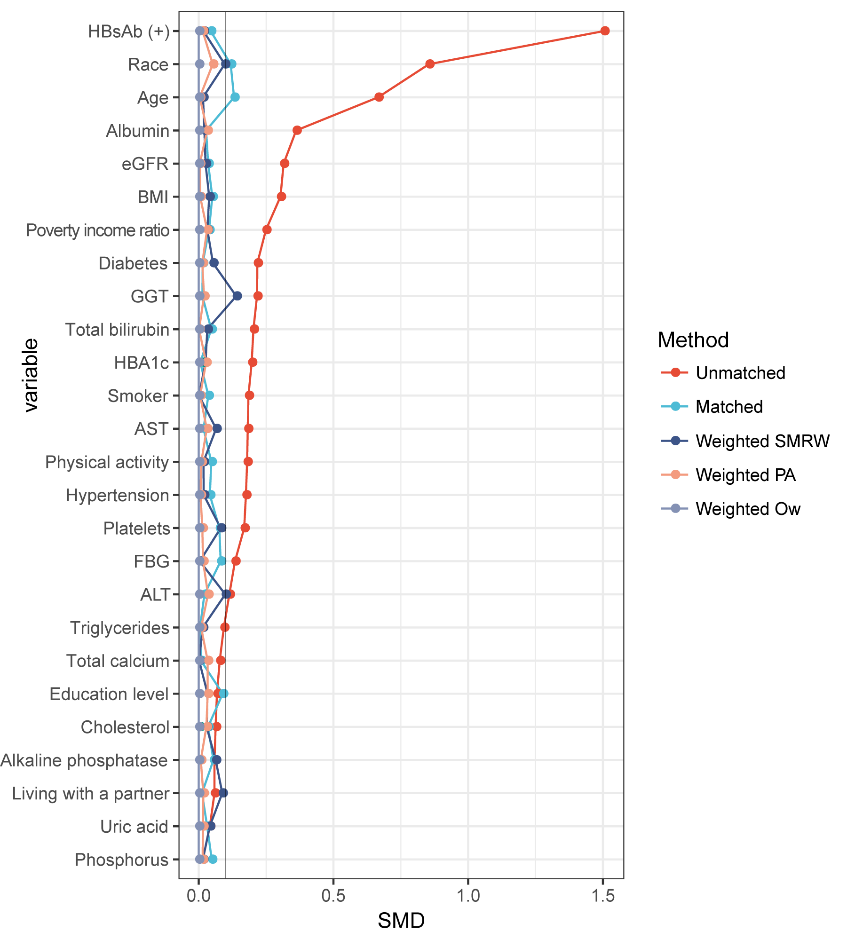


**Supplementary Figure 1.** Distribution of the standardized mean difference before and after propensity score matching

HBsAb, hepatitis B surface; BMI, body mass index; ALT, alanine transaminase; AST, aspartate transaminase; GGT, gamma-glutamyl transpeptidase; eGFR, estimated glomerular filtration rate; FBG, fasting blood-glucose; HBA1c, glycosylated hemoglobin; SMRW, the standardized mortality ratio weighting; PA, pairwise algorithmic; Ow, overlap weight; SMD, standardized mean difference


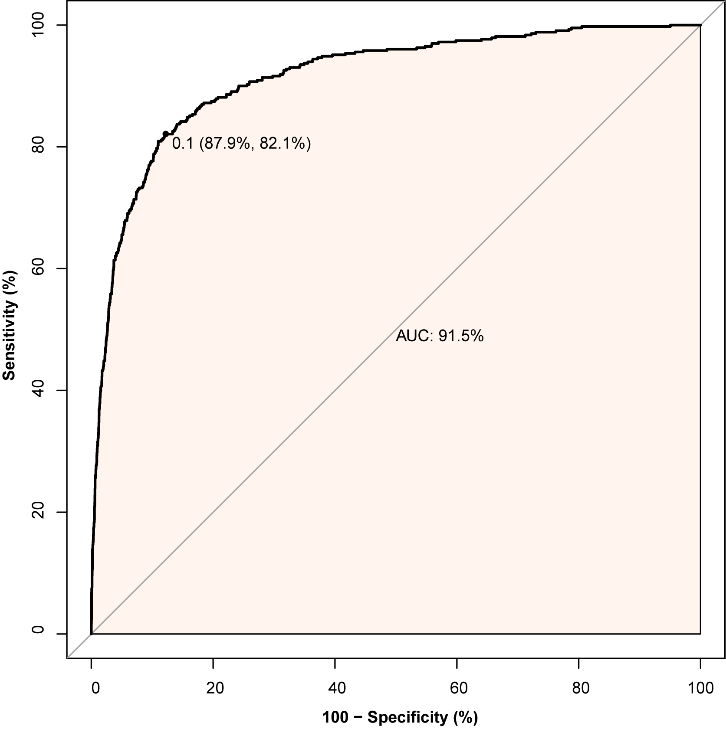


**Supplementary Figure 2.** Receiver operating characteristic curve of the propensity score model
